# Retraction: Artificial Liver Support System Combined with Liver Transplantation in the Treatment of Patients with Acute-on-Chronic Liver Failure

**DOI:** 10.1371/journal.pone.0221917

**Published:** 2019-08-27

**Authors:** The PLOS ONE Editors

Concerns have been raised that the transplants performed in the local context at the time of procedures reported in this article [1] may have involved organs/tissues procured from prisoners [2].

Details as to the donor sources were not reported in [1]. In response to journal inquiries the authors did not clarify this issue or the cause(s) of donor death, and they did not provide details as to the informed consent procedure used for donors. International ethics standards call for transparency in organ donor and transplantation programs and clear informed consent procedures including considerations to ensure that donors are not subject to coercion. The article [1] reports that donor livers were not obtained from executed prisoners, that donors and recipients provided written informed consent, and that the study had institutional ethics approval, but following journal requests the authors did not provide ethics approval documentation to support their claims.

In addition, the authors did not respond to inquiries about the availability of underlying data supporting this study.

Owing to the lack of documentation to demonstrate this study had prospective ethical approval, insufficient reporting, unresolved concerns around the source of transplanted organs and whether they included organs from prisoners, and in compliance with international ethical standards for organ/tissue donation and transplantation, the *PLOS ONE* Editors retract this article.

The corresponding author notified the journal that all authors disagree with the retraction. Xiao Xu, XL, QL, QW, ZL, LZ, SZ confirmed their disagreement, the other authors either could not be reached or did not respond directly.
